# N. B. Slayton

**Published:** 1859-10

**Authors:** W. M. Hunter


### N. B.—SLAYTON.

MESSRS. EDITORS:—I believe it is very generally known that I am opposed to patenting any thing that has for its object the alleviation of human suffering, even were the inventor entitled by law, to a patent—thinking as I do, that the unscrnpulous as well as the ignorant operator, takes thereby an advantage not consistent with the true dignity of a professional gentleman—much more am I opposed to the dabbling experimenter who fancies that he has discovered a great desideratum, and straightway makes an application for, and receives a patent for that which is perhaps of no practical utility, or of only limited application, and probably before used and thereby dedicated to the public long before the so claimed *inventor* had taken his bandanna from his pocket and wiped the dust off from his *specs* to *see into it.*

It is true that I can not prevent members of the profession from procuring patents, nor do I desire to do so, if they have a *valid* claim, however much I may be opposed to the policy. But I will not "stand at ease" when I know of the law being invoked to throw a halo of glory around a worthless or invalid process, as laid down on the usual *sheep skin* issued from the Patent Office, and granted by parties who know just about as much of the subject matter of it as does a bell-wether the price of putty—no, no, if we must have patents, let there be practical utility, novelty, and all the requirements of the law, and then will I hold my peace—and for this I am denounced as crazy.

> "That he is mad, 'tis true: 'tis true, 'tis pity;
> And pity 'tis, 'tis true; a foolish figure;
> But farewell it, for I will use no art.
> Mad let us grant him then."

Slayton's patent having been granted August 7th, 1859, let us quote the specification.

"The nature of my invention relates 1st the securing of art teeth upon plates of gold and silver *by means of an amalgam* of gold and silver, or both, combined with mercury. 2d. Forming by means of said amalgam an outer flange or rim which may constitute a step in which the base of the teeth may rest."

"The operation of securing teeth upon plates of gold or silver by means of my invention, may be described as follows:—

"The plate will be struck and fitted to the mouth, the articulation secured and the teeth ground to fit in the ordinary manner—the teeth will then be fitted to their places upon the plate, the outside of the teeth and plate will then be inserted in a preparation of asbestos and plaster of Paris *or other suitable preparation* for properly securing the teeth in their places. *The plate upon the back of the teeth* will then be well cleaned in order to remove all foreign substances, and a brass or silver wire brush, first dipped in a solution of nitrate of mercury and then in pure mercury will be passed over the plate at the points where the amalgam is desired to adhere until those points are perfectly silvered over. Gold or silver or both combined, properly prepared by precipitation or other mode with mercury, and well tritarated will be placed in a buckskin or other suitable sack and then in a vice, and all the mercury possible pressed out in that manner—the amalgam is then again triturated, and with proper instruments packed around the backs of the teeth, imbedding the hooked rod or wire and perfectly filling the interstices between the teeth, and forming the back as shown in fig. 2, the finish is made complete as no allowance is necessary for shrinkage. The inside or back of the teeth are now invested with the preparation before described, in such a manner as to cover all the amalgam—the said preparation will then be removed with a suitable sharp instrument from the outer edge of the plate, and such portion of the teeth as are to be covered by the flange or rim—the outer edge of the plate will then be treated in the manner above described, and the rim formed of the amalgam precisely similar to the backing upon the inside of the teeth as above set forth. The finish now being complete the outside of the plate and the rim formed as aforesaid will be again invested in the said preparation of asbestos and plaster of Paris, so as to completely cover all the amalgam, two slight perforations being made at either end of the row of teeth to permit the escape of the mercury; the whole will then be placed in an oven or other suitable vessel for the purpose, and subjected to a heat of at least seven hundred degrees, and kept there from two to four hours or until the mercury is entirely driven off! *the amalgam will then have adhered to the plate in such manner as to form par*

*of the same, and the teeth will be secured in the most perfect manner*, should greater strength be required (after this most perfect manner? W. M. H.) after the heating process, the amalgam may be washed with a solution of borax, and gold or silver solder applied; the whole will then be subjected to a heat sufficient to melt the solder which will flow freely through the cells vacated by the mercury, *thereby* forming a perfect union of the plate and amalgam (still amalgam after the evaporation of the mercury? W. M. H.) in one solid mass of metal." (Now, reader, look at the claims 1st and 2d, and tell me what unites the teeth to the plate, the amalgam or the solder?) Wonderful claimant, astute commissioner!

The above is a correct copy of Slayton's process from the Patent Office, and as Slayton with impudence almost unparalleled, in the Sept. number of the "Register" stated that in "no important point" did I describe his work, I will here transcribe a portion of my publication in the July number of the same.

I say,—" The process is simply this:—Having struck up your plates, ground, arranged and articulated your teeth in the usual way, flatten and bend the pins and invest the outside of the teeth and plate in a mixture of plaster and sand or asbestos, to retain them in their position; then prepare your amalgam of gold or silver (as the case may be) with mercury, either filings or precipitates may be used) triturate well, and press out as dry as you can with heavy pliers the free mercury—then with a blunt instrument pack the amalgam over the teeth down to the plate, to the fullness desired, filling every crevice under the teeth, &c.; then invest the inside of the plate (over the amalgam) with plaster and sand or asbestos, and cut from the outside whatever is necessary to expose the surface to be occupied by the rim; pack on the amalgam as before, and cover with plaster and sand, then cut two holes, one at each extremity of the jaw through the investient down to the amalgam, to allow the mercury to escape; then place in an oven of a stove or in any position where a heat of from 680 to 700 deg. can be applied for four or five hours, or sufficient time to evaporate all the mercury. After the mercury is all evaporated renew the investient, and if additional strength is required, cover the surface with a solution of borax, and flow solder over it, so that it will pass through the pores or cells down to the plate; then burnish and polish."

Now, dear reader, again, what do you think of the veracity of a man who can sit down and pen such an article after what I have put before you? He certainly did not expect to get a patent, or was ignorant that a copy of it could be had after it was issued. His offer of instruction, you see, was superfluous, for I had already given a clearer description than the patent specification. The merits of the patent I do not consider worthy of comment, as I said before, the only application of utility that can be made is the building up of a masticating surface, where the teeth are so short that strength enough can not be obtained in the mineral tooth, and then the work is backed and soldered in the usual way, the bearing or grinding surface being supplied with metal. Such work I have done years ago (and will do again when the case demands it) and it *entirely invalidates the patent.* Without the solder the work is not one of practical utility, as evidenced by my breaking Slayton's show jobs (which were not soldered) simply with my thumb and fingers—and this is the work that was to be palmed off upon the profession for a "*reasonable sum for an office right*," if they wished the *improvement!!!* Agents were getting ready to make their "tarnal fortin" by peddling the "wonder," and one hailing from Cincinnati had gone so far as to give up his business for the venture, notwithstanding what Slayton says. I feel half sorry that I prevented his following the vocation, for I think him much more eminently qualified for that business or the engineering of a steed horse than the practice of dentistry.

On the gutta percha question I have a few words to say. I consider it a *necessary* about the laboratory to restore the integrity of temporary cases, and is much better than the almost black article which I used before Slayton succeeded in giving it a color somewhat resembling the gums, which I now use in preference. I gave him a certificate that I believed it a good thing, *or at all events was going to try it*—and if he used it other than as above, I am very sorry that I should have been a party to the swindle.

That I claimed other than as above stated for my use of gutta percha, I give the "lie direct," and that it may be known how to make the article, it is simply necessary to knead the gutta percha made plastic in hot water, with zinc white, until it will take a red color with ordinary polishing rouge.

I confess to the profession and to Slayton that I have advertised him more than he deserves, but having "put my foot in it," I have been compelled to proceed, hoping that circumstances may not compel me soon again to resort to the mode of warfare practised by the Yahoo.

CINCINNATI, AUG. 14, 1859.

This is to certify that about four years ago Wm. M. Hunter made for me a set of artificial teeth with metallic masticating surfaces on gold plate, which has been worn with satisfaction to the present time. (Signed) JACOB GRAFF.

The end was accomplished by the use of amalgam, the mercury being driven off by heat and the pores filled with solder, which work I manipulated in Dr. Hunter's Laboratory.
JOS. DOUGHTY.

I examined the above at the time of making, and have likewise frequently used amalgam in the process of mounting teeth on platina plate for continuous gum work, in Dr. Hunter's Laboratory. GEO. W. KENDALL.

The above is only published for the purpose of preventing imposition upon the profession, not that it is very wonderful, although good. W. M. HUNTER.

